# 20(S)-Protopanaxadiol Inhibits Titanium Particle-Induced Inflammatory Osteolysis and RANKL-Mediated Osteoclastogenesis via MAPK and NF-κB Signaling Pathways

**DOI:** 10.3389/fphar.2018.01538

**Published:** 2019-01-18

**Authors:** Chenhao Pan, Haojie Shan, Tianyi Wu, Wei Liu, Yiwei Lin, Wenyang Xia, Feng Wang, Zubin Zhou, Xiaowei Yu

**Affiliations:** ^1^Department of Orthopedic Surgery, Shanghai Jiao Tong University Affiliated Sixth People’s Hospital, Shanghai, China; ^2^Department of Orthopedic Surgery, Shanghai Sixth People’s Hospital East Campus Affiliated to Shanghai University of Medicine and Health Sciences, Shanghai, China

**Keywords:** inflammation, bone resorption, MAPK signaling, NF-κB signaling, 20(S)-protopanaxadiol

## Abstract

Osteolysis is a principal reason for arthroplasty failure like aseptic loosening induced by Titanium (Ti) particle. It is a challenge for orthopedic surgeons. Recent researches show that 20(S)-protopanaxadiol can inhibit inflammatory cytokine release *in vitro*. This study aims to assess the effect of 20(S)-protopanaxadiol on Ti particle-induced osteolysis and RANKL-mediated osteoclastogenesis. Micro-CT and histological analysis *in vivo* indicated the inhibitory effects of 20(S)-protopanaxadiol on osteoclastogenesis and the excretion of inflammatory cytokines. Next, we demonstrated that 20(S)-protopanaxadiol inhibited osteoclast differentiation, bone resorption area, and F-actin ring formation in a dose-dependent manner. Moreover, mechanistic studies suggested that the suppression of MAPK and NF-κB signaling pathways were found to mediate the inhibitory effects of 20(S)-protopanaxadiol. In conclusion, 20(S)-protopanaxadiol may suppress osteoclastogenesis in a dose- dependent manner and it could be a potential treatment of Ti particle-induced osteolysis.

## Introduction

Total hip arthroplasty is a successful treatment strategy that improves quality of life for patients with end-stage rheumatic arthritis or other severe joint diseases. However, this procedure is accompanied by a possibility of aseptic loosening or fracture. Peri- implant osteolysis (PIO) by particle-induced inflammatory responses is considered to be the key pathophysiology factor of loosening (Lucas et al., 1998; Anderson et al., 2008).

Particulate wear debris is produced by biological or mechanical responses after implanting. With the widespread application of titanium alloy prosthesis, titanium (Ti)-particles, which can induce the recruitment of immune cells and osteoclasts to the bone-implant interface and lead to osteolysis, have become an important factor of PIO in metal-metal or metal-polyethylene hip arthroplasty (Markel et al., 2009; Ren et al., 2014). The immune cells, such as macrophages, release inflammatory cytokines like tumor necrosis factor (TNF)-α and interleukin (IL)-1β to establish a peri-implant inflammatory microenvironment (Bi et al., 2001; Keating et al., 2007). The inflammatory microenvironment was rich in macrophage colony-stimulating factor (M-CSF) and receptor activator of nuclear factor-κB ligand (RANKL), which promoted osteoclast precursors (OCPs) and osteoclast differentiation (Takahashi et al., 2003; Ren et al., 2008; Mao et al., 2016). It played a key role in the activation of downstream signaling molecules for macrophage and osteoclast maturation, such as mitogen-activated protein kinase (MAPK), and nuclear factor-κB (NF-κB) (Boyle et al., 2003; Feng, 2005; Yan et al., 2017). Hence, inhibiting the signaling pathways of osteoclasts might restrain the formation of osteoclasts, which could provide a potential treatment strategy for the inflammatory osteolytic diseases.

**FIGURE 1:**
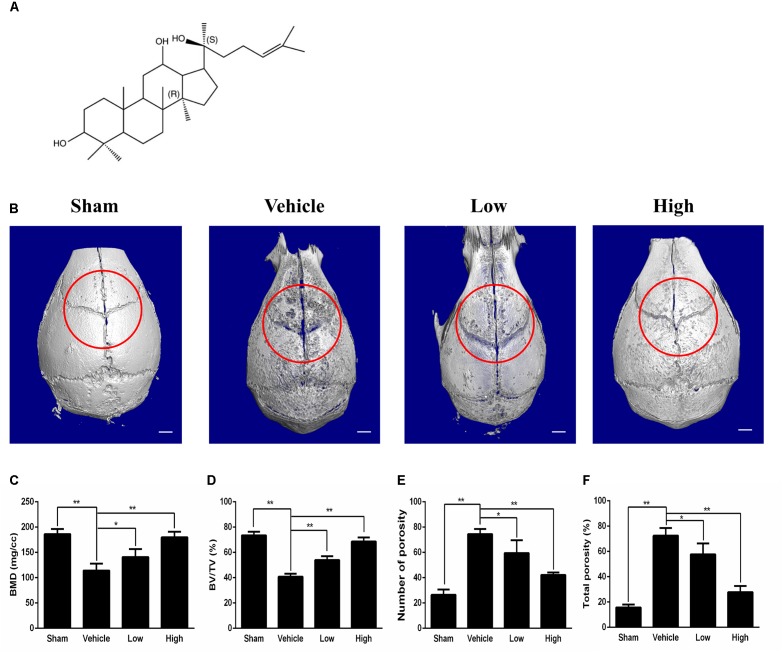
Micro-CT scanning analysis of bone loss for the effect of PPD on the murine calvarial osteolysis model. **(A)** Chemical structure of PPD. Molecular formula of C30H52O3 (≥ 97% purity) with an average molecular weight of 460.73. **(B)** Representative images of reconstructed 3D calvarias from each group. Graphic depiction of **(C)** bone mineral density (BMD, mg/cc), **(D)** bone volume/total volume (BV/TV, %), **(E)** number of pores and **(F)** total porosity within the ROI of each sample were measured. Significant differences between the groups were determined by ANOVA and Dunnett’s *t*-test. ^∗^*P* < 0.05 and ^∗∗^*P* < 0.01 compared with the vehicle group. Data are expressed as the means ± SD.

20(S)-protopanaxadiol (PPD) (Figure 1A) is an active extraction from ginseng (Liu J. et al., 2014). PPD has been used as a Chinese traditional medicine to exhibit wide pharmacological properties, such as anti-cancer, anti-diabetes, anti-fatigue and anti-inflammatory effects (Oh et al., 2015; Deng et al., 2017; Patel and Rauf, 2017; Bronsveld et al., 2018). PPD had an inhibitory effect on macrophages by inhibiting the PDK1/Akt pathway (Jeong et al., 2013). Park et al. reported the protection of PPD for the cerebral ischemia and other neuroinflammatory disorders (Park et al., 2012). Wu et al. suggested that PPD might attenuate the function of B cells and macrophages in arthritis rats (Wu et al., 2014). However, little is known about the anti-inflammatory effect of PPD on osteolysis. There is a hypothesis that PPD can suppress Ti particle- induced inflammation and osteolysis.

In this study, we aimed to (1) investigate the direct inhibitory effect of PPD on Ti particle-induced osteolysis *in vivo*, (2) investigate the effect of PPD on the expression of inflammatory factors *in vivo*, (3) find out the suitable dose of PPD that can regulate osteoclast formation and function with more detail, and (4) find out the potential signaling pathways involved in PPD inhibition of RANKL-induced osteoclast formation and bone resorption.

## Materials and Methods

### Media, Ti Particles and Reagents

20(S)-protopanaxadiol was taken from Sigma (≥ 97% purity, P0031, St. Louis, United States), dissolved in dimethyl sulfoxide (DMSO) and stored at −20°C. RAW264.7 cells were obtained from the Shanghai Cell Bank of the Chinese Academy of Science (Shanghai, China). Fetal bovine serum (FBS) and alpha modification of Eagles medium (α-MEM) were purchased from Gibco-BRL (Sydney, Australia). Recombinant murine M-CSF and RANKL were purchased from PeproTech (NJ, United States). Specific primary antibodies against NF-κB inhibitor alpha (IκBα) (#4814), p-IκBα (#9246), NF-κB p65 (#8242), phospho-NF-κB p-p65 (#3033), ERK1/2 (#4695), phospho-ERK1/2 (#4370), SAPK/JNK (#9252), phospho-SAPK/JNK (#4668), p38 (#8690), phospho-p38 (#4511), histone H3 (#4499), β-actin(#4967) and c-Fos (#4384) were purchased from Cell Signaling Technology (MA, United States). Antibodies against transforming growth factor activated kinase-1 (TAK1) (ab109526), phospho-TAK1(ab109404), NFATc1 (ab175134), were purchased from Abcam (Cambridge, United Kingdom). The Cell Counting Kit-8 (CCK8) was obtained from Dojindo Molecular Technology (Japan). The Prime Script RT reagent kit and SYBR1 Premix Ex TaqTM II were obtained from TaKaRa Biotechnology (Otsu, Shiga, Japan). Ti particles (99.99% purity, diameter ranging from 1 to 3 μm) were obtained from the Johnson Matthey Company (MA, United States).

### Ti Particle-Induced Calvarial Osteolysis Model

All experimental procedures were performed in strict accordance with the guidelines of the Institutional Review Board of Animal Care Committee of Shanghai Jiao Tong University Affiliated Sixth People’s Hospital (Ethical Approval Number: 2018-0094). Mouse calvarial model was created as described by previous studies (Liu X. et al., 2014; Hu et al., 2017). Ti particles were submersed in 75% ethanol for 48 h. They were then sterilized at 121.3°C for 1 h, and resuspended in sterile PBS at a concentration of 300 mg/mL. 28 male, 8-week-old C57BL/6 mice assigned randomly to 4 groups: PBS control (sham), Ti particles (vehicle), Ti particles with 2 mg/kg/day (low) and 5 mg/kg/day (high) PPD, which were obtained from Shanghai Sixth People’s Hospital under specific pathogen-free (SPF) conditions. 0.1 mL of Ti particles were embedded under the periosteum at the middle suture of the calvaria as described by previous studies (Liu X. et al., 2014; Hu et al., 2017). Next, PPD or PBS was injected into the periosteum every day for 14 days. No adverse effects or mortality occurred during the experiment. At the end of the course, the calvarias were excised and fixed in 4% (w/v) paraformaldehyde for further experiments.

### Micro-CT Scanning

The fixed calvarias were analyzed using a micro-CT (Skyscan, 1072; Skyscan, Aartselaar, Belgium). The scanning protocol was set at an isometric resolution of 9 mm, and the X-ray energy settings were 80 kV and 80 mA. After reconstruction, the region of interest (ROI) around the intersection of the coronal and sagittal midline suture was selected for analyses, including the bone mineral density (BMD), bone volume/tissue volume (BV/TV), the number of pores and the percentage of total porosity (Kauther et al., 2013).

### Histological and Immunochemical Analysis

Samples were decalcified in 10% (w/v) ethylenediaminetetraacetic acid (EDTA) for 3 weeks, followed by paraffin embedding. Hematoxylin and eosin (H&E) staining, TRAP staining and immunohistochemistry staining were performed. A commercial tartrate-resistant acid phosphatase kit (#387A, Sigma-Aldrich) was used for TRAP staining as described previously (Ping et al., 2017). The number of claret-red cells counted as TRAP-positive osteoclasts and the percentage of osteoclasts per bone surface (OcS/BS %) from five consecutive slices were calculated by two independent observers. As for immunohistochemistry staining of TNF-α, IL-1β, calvarial sections were incubated with rabbit anti-mouse primary antibodies (1:1000; purchased from Abcam) at 4°C for overnight (Ping et al., 2017). Sections were then incubated with secondary antibodies for 1 h prior to counterstain with hematoxylin. The positive cells were calculated from five consecutive slices by two independent observers.

### Bone Marrow-Derived Macrophages (BMDMs) Isolation and Osteoclasts Culture

Bone marrow-derived macrophages were isolated from the long bone marrow of 5 weeks old C57BL/6 mice (Li et al., 2018). Briefly, bone marrow cells were flushed out from the femur and tibia and cultured in α-MEM with 10% (v/v) FBS, 1% (w/v) penicillin/streptomycin and 10 ng/mL M-CSF for 24 h. The non-adherent cells were harvested and cultured in complete α-MEM (α-MEM containing 10% (v/v) heat inactivated FBS, 2 mM L-glutamine, 100 U/mL penicillin/streptomycin, and 30 ng/mL M-CSF) for an additional 3–4 days. Then nearly 90% confluence, the cells were collected. The Raw264.7 cell line was cultured in α-MEM supplemented with 10% (v/v) FBS, 2 mM L-glutamine and 100 U/mL penicillin/streptomycin. All the cell cultures were maintained at 37°C in a humid environment with 5% (v/v) CO_2_.

### Cell Cytotoxicity Assay

The effect of PPD on BMDMs cell viability was determined by the CCK8 assay. BMDMs plated in 96-well plates at a density of 1 × 10^4^ cells/well in triplicate were cultured in complete α-MEM medium containing different concentrations of PPD (0, 1.25, 2.5, 5, 10, 20, 40, and 80 μM) for 48 h. Then, 10 μL CCK-8 was added to each well of the plates incubated at 37°C for an additional 2 h. The optical density (OD) was observed at a wavelength of 450 nm with a microplate eader (Multiskan MK3; Thermo Fisher Scientific, United States). The half-maximal inhibitory concentration (IC50) was calculated using GraphPad Prism version 5.0c (San Diego, CA, United States).

### TRAP Staining

Bone marrow-derived macrophages were seeded into a 6-well plate at a density of 8 × 10^5^ cells/well with complete α-MEM medium, M-CSF (30 ng/mL), RANKL (100 ng/mL), and PPD (0, 1.25, 2.5, or 5 μM) for 7 days. The culture medium was replaced every 2 days. The cells were fixed with 4% (w/v) paraformaldehyde for 20 min and stained for TRAP by the Diagnostic Acid Phosphatase kit. TRAP-positive cells with more than three nuclei were considered as osteoclast-like (OCL) cells. The average size and number of osteoclasts were calculated for each group in triplicate for further analysis.

### Resorption Pit Formation Assay

Corning Osteo Assay Surface 24-well plates were used to determine the inhibitory effect of PPD on the bone resorption function of osteoclasts. BMDMs were plated in it and incubated with complete α-MEM medium, M-CSF (30 ng/mL), RANKL (100 ng/mL). After 4 days, when the formation of osteoclasts was observed, PPD (0, 1.25, 2.5, or 5 μM) was added for an additional 2 days. The cells were then removed by an ultrasonic generator. The three random fields of view at the bottom of the wells were photographed through the Olympus DP70 inverted microscope (Japan), and three random fields of view were analyzed for each well. The percentage of the bone resorption area was analyzed for each well using ImageJ software.

### F-Actin Ring Staining

Bone marrow-derived macrophages seeded into a 24-well plate were treated with complete α-MEM medium, M-CSF (30 ng/mL), RANKL (100 ng/mL) and PPD (0, 1.25, 2.5, or 5 μM) for 7 days. Then osteoclasts were fixed with 4% (w/v) paraformaldehyde for 20 min and then treated with 0.1% (v/v) Triton X-100 for 15 min. Cells were incubated with rhodamine-conjugated phalloidin (Invitrogen Life Technologies, Grand Island, NY, United States), diluted in 1% (w/v) bovine serum albumin (BSA) in phosphate-buffered saline (PBS) for 30 min at room temperature. Cells were then incubated with DAPI for 5 min. Fluorescence images were taken and were analyzed using ImageJ software.

### Western Blotting Analysis

RAW264.7 cells and BMDMs were seeded into a 6-well plate. After treatment with PBS or PPD for 2 h, the RAW264.7 cells were stimulated by RANKL (100 ng/mL) for 0, 5, 15, or 30 min. BMDMs were cultured with 0 or 5 μM PPD, M-CSF (30 ng/mL) and RANKL (100 ng/mL) for 0, 1, 3, and 5 days. Next, the total protein and nuclear protein were extracted with radioimmunoprecipitation assay (RIPA) lysis buffer containing PMSF (EpiZyme, and phosphatase inhibitors (EpiZyme, Shanghai) or with a nuclear protein extraction commercial kit (Beyotime, China) according to the manufacturer’s instructions. The concentration of protein was determined with a bicinchoninic acid (BCA) protein assay kit (Beyotime, China) according to the manufacturer’s instructions. Equal amounts of the protein (40 μg) lysates were separated using 8 or 10% (w/v) sodium dodecylsulfate-polyacrylamide gel electrophoresis (SDS-PAGE) and electroblotted onto polyvinylidene fluoride (PVDF). After blocking with 5% (w/v) skim milk solution for 1.5 h, the PVDF membranes cut into strips were incubated with primary antibodies against TAK1, p-TAK1, p38, p-p38, JNK, p-JNK, ERK, p-ERK, IκBα, p-IκBα, NF-κB, p-NF-κB, c-fos, NFATc1, β-actin and histone H3 at 4°C overnight. The membranes were then incubated with horseradish peroxidase-conjugated secondary antibodies (1:5000) for 1 h. The antibody reactivity was subsequently visualized using enhanced chemiluminescence reagent (Beyotime, Shanghai) prior to detection by ChemiDoc CRS imaging system (Bio-Rad, United States).

### NF-κB Luciferase Reporter-Gene Assay

RAW264.7 cells were transfected with a p-NF-κB-TA-Luc luciferase reporter construct (Kammerer et al., 2003; Wu et al., 2015). Briefly, cells were plated in 24-well plates at a density of 5 × 10^4^ cells/well. After adding 5 μL Lipofectamine^®^ 2000 (Thermo Fisher Scientific, United States), p-NF-κB-TA-Luc was diluted in complete α-MEM medium, then additional 50 μL Lipofectamine^®^ 2000 was added. The complex was incubated for 5 min at room temperature and added into cells for 2 days to get transfected cells. After incubated additional 24 h, the transfected cells were treated with PPD (0, 1.25, 2.5, and 5 μM) for 2 h, and then incubated with 100 ng/mL RANKL for 12 h. After lysing cells, the luciferase activity was measured using the Promega Dual-Luciferase Reporter Assay System.

### Statistical Analysis

All data are presented as mean ± SD. Statistical analysis was performed by combining one-way analysis of variance (ANOVA), Dunnett’s or Paired Student’s *t*-test using the SPSS 19.0 software (Chicago, IBM, United States). Statistical differences were considered to be significant only if ^∗^*P* < 0.05 or ^∗∗^*P* < 0.01.

## Results

### PPD Inhibited Ti Particle-Induced Osteolysis and the Release of Inflammatory Factors *in vivo*

Ti particle-induced osteolysis modal was constructed to clarify the efficacy of PPD. Micro-CT scanning and 3D reconstruction illustrated that Ti particles led to dramatic surface erosion, but this was suppressed by treatment with PPD (Figures 1B–F). The bone parameters quantified showed that BMD of the Vehicle group decreased by 78.3%, and total porosity increased by 181.7% compared with Sham group. Compared with Vehicle group, BMD increased by 23.3 and 57.6% in the low and high doses group respectively, and total porosity decreased by 20.3 and 61.7% respectively (Figures 1C,F). Further quantification, PPD significantly increased the BV/TV and decreased the number of pores (Figures 1D,E).

**FIGURE 2:**
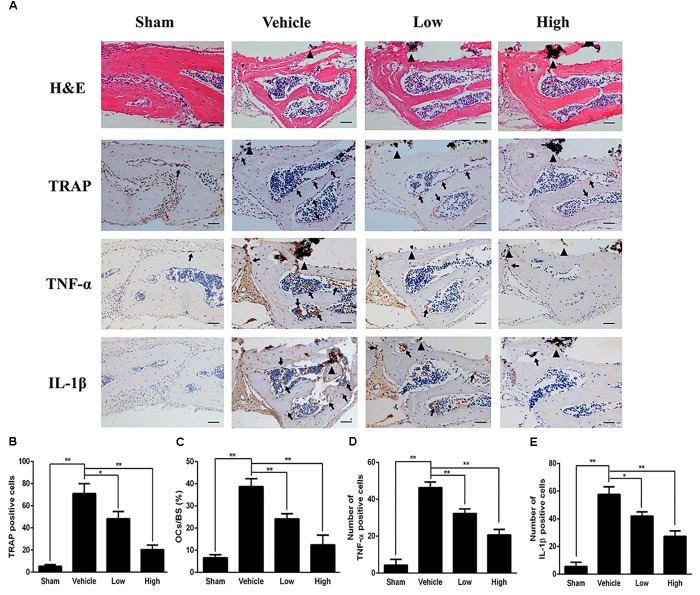
PPD prevents titanium particle-induced osteolysis and inhibits the expression of pro-inflammatory cytokines *in vivo*. **(A)** Histological images of HE-stained, TRAP-stained calvarium sections and immunohistochemical staining of tumor necrosis factor (TNF)-α, interleukin (IL)-1β; Calculated parameters **(B)** of the number of TRAP-positive cells, **(C)** ratio of osteoclast surface to bone surface (OcS/BS, %) **(D)**, the number of TNF-α cells and **(E)** the number of IL-1β cells were measured within the ROI of each sample. Significant differences between the groups were determined by ANOVA and Dunnett’s *t*-test. ^∗^*P* < 0.05 and ^∗∗^*P* < 0.01 compared with the vehicle group. Black triangles indicate Ti particles, black arrows indicate osteoclasts. Data are expressed as the means ± SD. Scale bar = 50 μm.

Histological and histomorphometric analysis further confirmed that PPD inhibited Ti particle-induced bone loss. H&E staining illustrated that bone destruction was suppressed in the PPD treated group, which differed from the vehicle group where osteolysis occurred typically (Figure 2A). TRAP staining with quantification showed that the number of osteoclasts in the vehicle group was increased. The number of osteoclasts and OcS/BS were both significantly reduced in the Low and High group (Figures 2A–C). Furthermore, the immunohistochemical staining revealed that there were numerous TNF-α, IL-1β positive cells in vehicle group (Figure 2A). In comparison, the number of TNF-α, IL-1β positive cells was much lower in both low and high dose groups (Figures 2D,E). Collectively, these findings revealed that PPD inhibited Ti particle-induced osteolysis and decreased the level of inflammatory factors *in vivo*.

### PPD Inhibits RANKL-Induced Osteoclast Formation in a Dose-Dependent Manner Without Affecting Cell Viability

**FIGURE 3:**
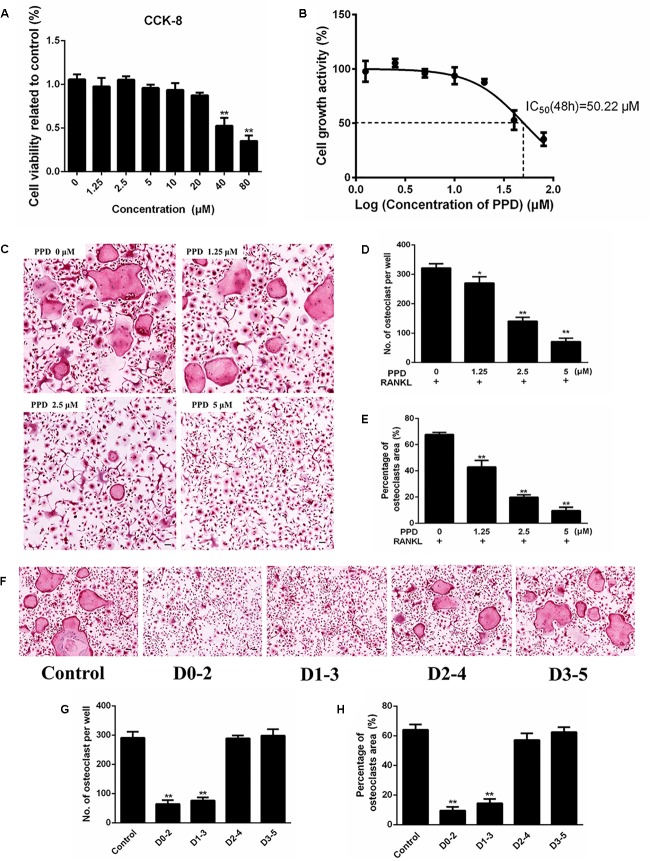
PPD inhibited RANKL-induced osteoclast formation without cytotoxic. **(A)** Cell viability was detected by a CCK-8 assay, and the results were normalized to the control group (i.e., the group without PPD treatment). **(B)** The half-maximal inhibitory concentration (IC50) was determined by GraphPad Prism. **(C–E)** BMDMs were stimulated with RANKL (100 ng/mL), M-CSF (30 ng/mL) and the indicated concentrations of PPD for 7 days; then, the cells were fixed and subjected to TRAP staining. The number and percentage of TRAP-positive cells were calculated. **(F–H)**. BMDMs were incubated in media containing 100 ng/mL RANKL and 30 ng/mL M-CSF with PPD (5 μM) from day 0 to 2, from day 1 to 3, from day 2 to 4 or from day 3 to 5, respectively. All BMDMs were incubated for 7 days. TRAP staining was performed to analyze the number and percentage of osteoclasts. Data are representative of at least three independent experiments with similar results. Significant differences between the groups were determined by ANOVA and Dunnett’s *t*-test. ^∗^*P* < 0.05 and ^∗∗^*P* < 0.01 compared with the control group. Data are expressed as the means ± SD. Scale bar = 100 μm.

CCK-8 was used to assess the cytotoxicity of PPD. The result illustrated that the IC50 value of PPD was 50.22 μM at 48 h (Figures 3A,B). BMDMs continued normal growth at doses up to 20 μM. The lower doses of PPD did not influence the viability of BMDMs. Further BMDMs were incubated with M-CSF (30 ng/mL), RANKL (100 ng/mL), and PPD (0, 1.25, 2.5, and 5 μM) for 7 days. BMDMs differentiated into TRAP-positive OCLs. However, the number of TRAP-positive OCLs exposed to PPD significantly decreased after TRAP staining in a dose-dependent manner (Figure 3C). The formation of osteoclasts was suppressed by approximately a half with treatment of 2.5 μM PPD (Figures 3D,E). In order to determine the effect of PPD on the stage of osteoclast differentiation, PPD interventions were divided into different time intervals. On Day 7, osteoclast formation was significantly suppressed by PPD added from Day 0 to 2 and from Day 1 to 3 group, while there was no significant difference after PPD added from Day 2 to 4 and from Day 3 to 5 group (Figures 3F–H). Therefore, PPD likely has a dose-dependent characteristic effect on inhibition of RANKL-induced osteoclast formation without cytotoxicity.

### PPD Inhibited Osteoclastic Bone Resorption and F-Actin Ring Formation

**FIGURE 4:**
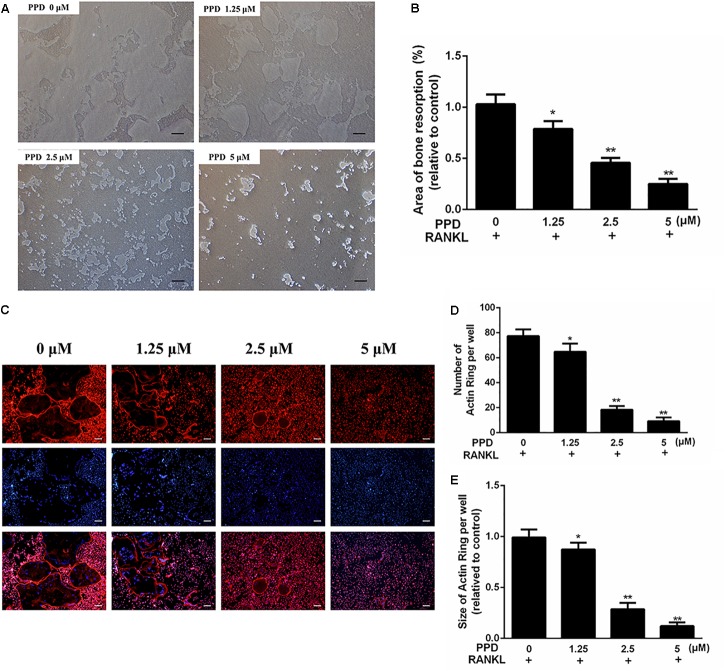
PPD inhibited the bone resorption area and F-actin ring formation during *in vitro* osteoclastogenesis **(A,B)**. BMDMs were inoculated onto the Corning Osteo Assay Surface 24-well plates and were cultured in differentiation medium for 4 days; then, various concentrations of PPD were added, and cells were incubated for two additional days. The area eroded by osteoclasts was quantified by ImageJ software **(C–E)**. BMDMs were treated with the indicated concentration of PPD in the presence of RANKL (100 ng/mL) and M-CSF (30 ng/mL) until mature osteoclasts appeared at day 6; then, the cells were stained with phalloidin and DAPI. Fluorescent F-actin rings were visualized under a microscope. The number and size of F-actin-positive cells was quantified by ImageJ software. Data are representative of at least three independent experiments with similar results. Significant differences between the groups were determined by ANOVA and Dunnett’s *t*-test. ^∗^*P* < 0.05 and ^∗∗^*P* < 0.01 compared with the control group. Data are expressed as the means ± SD. Scale bar = 200 μm.

As the differentiation of osteoclasts was obviously damaged by PPD, the function of osteoclast bone resorption which existed in osteolysis might also be inhibited. To confirm this, the osteoclastic bone resorption was performed by Corning Osteo Assay Surface 24-well plates. The bone resorption area decreased after treatment, compared to the control group (Figures 4A,B).

To investigate the effects of PPD on F-actin ring which was a precondition for bone resorption during osteoclastogenesis, F-actin ring of osteoclasts was stained with phalloidin (Figure 4C). The PPD interventions drastically suppressed F-actin ring morphology and formation. After PPD treatment, both the number and size of F-actin rings were inhibited with dose-dependent effects (Figures 4C–E). These findings suggested that PPD treatment impaired F-actin ring formation and bone resorption *in vitro*.

### PPD Suppressed RANKL-Induced Activation of MAPK and NF-κB Signaling Pathways

**FIGURE 5:**
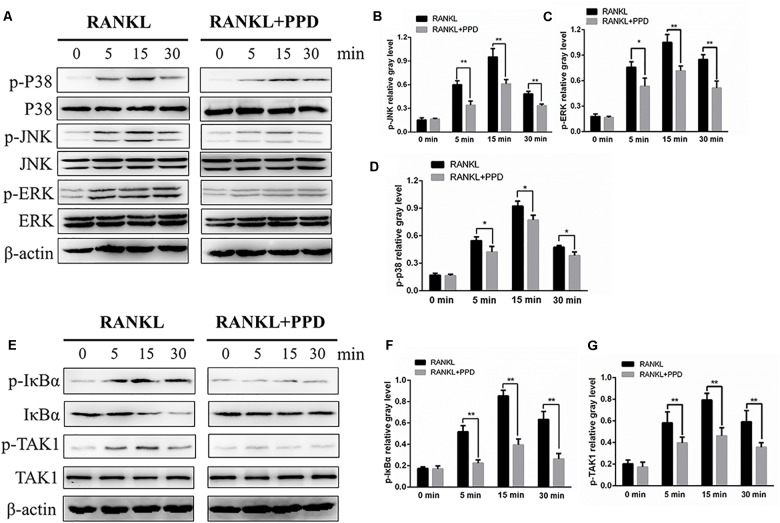
PPD inhibited osteoclast differentiation by specifically impairing RANKL-induced MAPK cascades and the NF-κB pathway. **(A,E)** RAW264.7 cells were treated with or without 5 μM PPD for 2 h and then treated with 100 ng/mL RANKL for the indicated periods. Cell lysates were analyzed using western blotting with specific antibodies against phospho-p38, p38, phospho-ERK1/2, ERK1/2, phospho-JNK1/2, JNK1/2, phospho-IκBα, IκBα, phospho-TAK1, TAK1, and β-actin. **(B–D)** The gray levels corresponding to phosphorylation of the indicated proteins were quantified and normalized relative to β-actin using Image J for p-p38, p-JNK, and p-ERK, **(F,G)** The gray levels corresponding to phosphorylation of the indicated proteins were quantified and normalized relative to β-actin using Image J for p-TAK1 and p-IκBα. Significant differences between the groups were determined by paired Student’s *t*-test. ^∗^*P* < 0.05 and ^∗∗^*P* < 0.01 compared with the control group.

**FIGURE 6:**
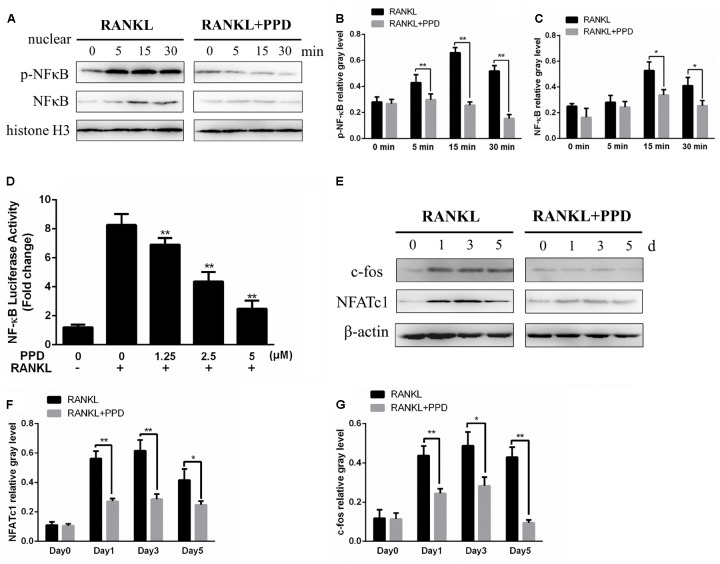
PPD inhibited osteoclast differentiation by the NF-κB pathway. **(A–C)** RAW264.7 cells were treated with or without 5 μM PPD for 2 h and then treated with 100 ng/mL RANKL for the indicated periods. Nuclear protein was isolated for western blot analysis. Cell lysates were analyzed using western blotting with specific antibodies against NF-κB and p-NF-κB were quantified and were normalized to H3 using ImageJ software. **(D)** RAW264.7 cells that had been stably transfected with an NF-κB luciferase reporter construct were treated with the indicated concentrations of PPD for 1 h, followed by incubation in the absence or presence of RANKL for 8 h. Luciferase activity was measured using the Promega Luciferase Assay System. All experiments were performed at least 3 times; **(E)** BMDMs were cultured with 30 ng/mL M-CSF and 100 ng/mL RANKL with or without 5 μM PPD for 0–5 days. Cell lysates were prepared and analyzed using western blotting. **(F,G)** The gray levels corresponding to the indicated proteins were quantified and normalized relative to β-actin using Image J for c-fos and NFATc1. Significant differences between the groups were determined by paired Student’s *t*-test. ^∗^*P* < 0.05 and ^∗∗^*P* < 0.01 compared with the control group.

To clarify the mechanism of inhibition of osteoclast formation and function after PPD interventions, western blotting and a dual-luciferase reporter assay were used. RAW264.7 cells line were incubated with RANKL (100 ng/mL) for 0, 5, 15 or 30 min in the presence or absence of PPD to investigate MAPK and NF-κB signaling pathways including p-p38, p38, p-ERK1/2, ERK1/2, JNK, p-JNK, p-IκBα and IκBα. The phosphorylation of p38, JNK1/2, and ERK1/2 peaked within 15 min after RANKL stimulation. Phosphorylation within MAPK and NF-κB signaling pathways significantly decreased after pretreatment with PPD (Figures 5A–D). As shown in Figures 5A–D, the whole process of phosphorylation of ERK1/2, JNK1/2 and p38 was inhibited. The data suggested that PPD inhibited the MAPK signaling pathway. Figures 5E,F. suggested that PPD also inhibited IκBα phosphorylation during osteoclast- ogenesis, which meant that the NF-κB signaling pathway was inhibited. As a unique endogenous protein inhibiting NF-κB activation, both the phosphorylation and degradation of IκBα are required for NF-κB activation and translocation. The peak of NF-κB activation and phosphorylation in the control group occurred in 30 min after RANKL stimulation, but reduced sharply if pretreated with PPD (Figures 6A–C). Moreover, the whole process of the phosphorylation of TAK1 which could activate both MAPK and NF-κB pathways was inhibited (Figures 5E,G). To further confirm that PPD inhibited NF-κB activation, the luciferase assay was performed with PPD (0, 1.25, 2.5, or 5 μM) (Figure 6D). NFATc1 and c-fos, which are long-term NF-κB signaling ways, play a key role in osteoclastogenesis (Asagiri et al., 2005; Huang et al., 2006; Li et al., 2018). Hence, the protein level of NFATc1 and c-fos was tested in the presence of 5 μM PPD or absence of PPD for 0, 1, 3, and 5 days. The results showed that c-fos and NFATc1 were inhibited by PPD in a time- and dose-dependent manner (Figures 6E–G).

## Discussion

Ti particle-induced osteolysis is a main cause of arthroplasty failure, which shortened prosthesis life from 15–20 to 10 years (Abu-Amer et al., 2007), often resulting in an expensive and complicated revision operation (Bozic et al., 2009). Although the technology and materials involved in orthopedic surgery have been developed, wear particles production from the interface is still inevitable. It is necessary to find out a medication for osteolysis. The inflammatory cytokines, which induce the activation of osteoclasts, are increased by wear particles and this can lead to osteolysis through the imbalance of osteoblast and osteoclast activity. Therefore, targeting the formation of osteoclasts is considered to be a practicable therapeutic strategy.

PPD is the main natural compound in the ginseng. In recent years, some researches showed that it had a potential anti-inflammatory effect (Lee et al., 2012; Yayeh et al., 2012). PPD has also been used in bone biology. RAW 264.7 differentiation was inhibited after PPD treatment by signal transducer and activator of transcription 3 (STAT3) (Cong et al., 2017). Otherwise, it was reported that the proinflammatory factor release of RAW 264.7 decreased after PPD treatment (Yayeh et al., 2012). Lee et al. demonstrated that PDD promoted bone morphogenetic protein-2 (BMP-2) and Runt-related transcription factor 2 (Runx2) gene expression (Siddiqi et al., 2014). Therefore, we wanted to confirm whether it could restrain osteolysis by inhibiting the differentiation of osteoclasts.

In this study, we demonstrated that PPD inhibited Ti particle-induced osteolysis through the suppression of MAPK and NF-κB signaling pathways. A Ti particle-induced murine calvarial bone loss model was formed to affirm the efficiency of PPD *in vivo*. Osteolysis was obvious in Ti-embedded animals, while it was significantly suppressed in the PPD-treated groups. H&E and TRAP staining showed that PPD reduced the number of TRAP-positive multinucleated osteoclasts. Moreover, there were less macrophages in the PPD group than in vehicle group, which suggested that PPD may restrain bone erosion. On the other hand, the immunohistochemistry staining of TNF-α and IL-1β was performed, and as expected TNF-α and IL-1β positive cells decreased, suggesting that PPD prevented Ti particle-induced osteolysis partly by inhibiting TNF-α and IL-1β secretion.

Furthermore, osteoclast formation was inhibited without cytotoxic effects *in vitro*. The F-actin ring of osteoclasts, which was the prerequisite for bone resorption (Weigert et al., 2009), was dramatically suppressed after PPD treatment. Bone resorption assays illustrated that the number of osteoclasts and area of their bone resorption pits *in vitro* decreased after PPD treatment. These effects of PPD on the inhibition of osteoclast formation and function were characterized by a dose-dependent manner. To understand the mechanism of PPD-induced inhibition, the exploration of RANKL-related signaling pathways was performed. Our findings demonstrated that NF-κB activation and MAPK phosphorylation were inhibited by PPD at the protein level. Previous studies indicated that the MAPK signaling pathway played an important role in osteoclast development downstream of RANK signaling (Lee et al., 2010; Stevenson et al., 2011; Dhanya et al., 2017). The activation of the NF-κB pathway is also an important part of osteoclast development (Takaesu et al., 2001; Keating et al., 2007). Previous studies found that in NF-κB knockout mice osteoclasts failed to form (Franzoso et al., 1997; Iotsova et al., 1997). Associated with the inhibitory effect of p-IκBα, the activation of NF-κB proteins was suppressed after the intervention of PPD in this study. Mitogen-activated protein 3 kinase 7 (MAP3K7), also known as TAK1, is an enzyme that can activate both MAKB and NF-κB pathways (Takaesu et al., 2001; Kong et al., 2017). We also found that p-TAK1 was suppressed after PPD treatment, which might explain why both MAPK and NF-κB signaling pathways were inactivated. Moreover, NFATc1, known as a key transcription factor of osteoclast formation, is a downstream target gene of NF-κB. NFATc1 auto-amplified after NF-κB translocation and recruitment to the NFATc1 promoter (Asagiri et al., 2005; Yamashita et al., 2007). The activity of c-fos was a prerequisite for the induction and translocation of NFATc1, regulated by MAPK signaling pathway (Lucas et al., 1998; Takayanagi et al., 2002; Matsumoto et al., 2004; Huang et al., 2006; Yamashita et al., 2007). As we hypothesized, PPD inhibited the activation of NF-κB and MAPK phosphorylation, accompanied with the c-fos and NFATc1 decrease.

Here we provide a potential new usage of PPD for the treatment of osteolysis. It means that PPD may inhibit Ti particles-induced PIO. The therapeutic dose of PPD (2.5 μM *in vitro* and 5 mg/kg/day *in vivo*) was lower than other compounds with similar effects on osteolysis toGö6983 (Feng et al., 2018), enoxacin (Liu X. et al., 2014), Flavopiridol (Hu et al., 2018) and so on. Compared to other compounds, besides its low therapeutic dose, the potential anti-cancer and anti-diabetes effects of PPD made it possible to restrain bone loss in osteolytic tumors or diabetic osteoporosis (Deng et al., 2017; Patel and Rauf, 2017; Bronsveld et al., 2018). However, there are several limitations to our study. Firstly, this osteolysis model cannot perfectly replicate the PIO because of the differences in moving function, joint structure and arthroplasty insertion (von Knoch et al., 2004). Although titanium prosthesis is widely used, the incidence of prosthesis caused by UHMWPE is higher due to its easier abrasion (Hirakawa et al., 1996; Baumann et al., 2005; Ping et al., 2017). But, they have a similar importance in osteolysis formation (von Knoch et al., 2004). Secondly, in this study, PPD was injected into the periosteum once a day. The bioavailability might be 80%, however, there is a lack of direct evidence of bioavailability. More details about the bioavailability and administration of PPD are needed. Thirdly, despite the inhibition of the NF-κB and MAPK phosphorylation, the PPD-targeted protein and its inhibitor has not been determined. On the other hand, the correlation between the reduction of inflammation and osteoblasts or bone marrow stromal cells was not investigated, which is an indirect mechanism of osteoclastogenesis. Thus, further studies are needed.

To conclude, our study demonstrated that PPD had inhibitory effects on Ti particle-induced osteolysis *in vivo*. We also knew that MAPK and NF-κB signaling pathways played a critical role in the effects of PPD on inhibition of inflammatory bone resorption and osteoclastogenesis. PPD may represent a potential agent for the treatment of PIO. Furthermore, more researchers may be inspired to concentrate on the potential anti-inflammatory effects of PPD on other orthopedic diseases.

## Author Contributions

CP, HS, and TW designed and performed the experiments. WL, YL, and WX assisted in the experiments. YL, WX, FW, CP, and WL analyzed the data. CP and HS wrote the manuscript. ZZ and XY revised the manuscript and gave important advice to the study.

## Conflict of Interest Statement

The authors declare that the research was conducted in the absence of any commercial or financial relationships that could be construed as a potential conflict of interest.
